# Supplementary material for: Combining the Classification and Pharmacophore Approaches to Understand Homogeneous Olfactory Perceptions at Peripheral Level: Focus on Two Aroma Mixtures
Source: Molecules. 2023 May 11;28(10):4028. doi: 10.3390/molecules28104028 (PMC10221229; doi:10.3390/molecules28104028)
Supplement: Supplementary file 1 [file molecules-28-04028-s001.zip › Figure S2.pdf]

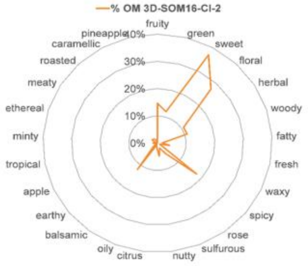

(a)

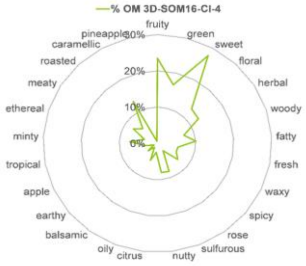

(b)

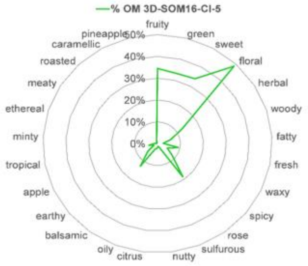

(c)

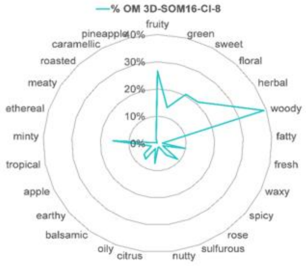

(d)

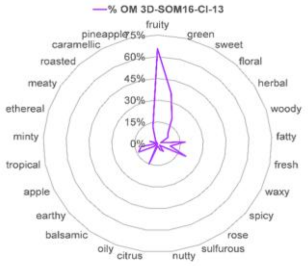

(e)

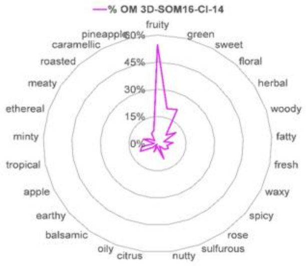

(f)

Figure S2. Radar charts of the distribution of the %OM values for the 25 most frequent odor notes across the 6 clusters SOM16 that contain the molecules of interest. (a) SOM16-Cl-2, (b) SOM16-Cl-4, (c) SOM16-Cl-5, (d) SOM16-Cl-8, (e) SOM16-Cl-13, (f) SOM16-Cl-14.
