# Supplementary material for: Combining the Classification and Pharmacophore Approaches to Understand Homogeneous Olfactory Perceptions at Peripheral Level: Focus on Two Aroma Mixtures
Source: Molecules. 2023 May 11;28(10):4028. doi: 10.3390/molecules28104028 (PMC10221229; doi:10.3390/molecules28104028)
Supplement: Supplementary file 1 [file molecules-28-04028-s001.zip › Table S3.pdf]

Table S3. Details of the PHASE-generated hypothesis from the subset composed of the mixture components.

Subset	Hypothesis	Phase Hypo Score	EF1%	BEDROC160.9	Ranked Actives
V-IA-F-EA-bI-bD-s	AHH_1	0.62	33.53	0.39	3
V-IA-F-s	AAR_1	0.78	66.87	0.50	2
	AAR_2	0.51	33.43	0.39	2
	AAH_1	0.47	33.43	0.39	2
WL-IA-s	AAH_1	1.21	100.20	1.00	2
	AAH_2	1.21	100.20	1.00	2
	AAH_3	1.20	100.20	1.00	2
	AHH_2	1.10	50.10	0.63	2
	AHH_5	1.01	50.10	0.56	2
	AAH_4	1.01	50.10	0.55	2
	AHH_1	0.90	50.10	0.54	2
	AHH_3	0.88	50.10	0.54	2
	AHH_4	0.87	50.10	0.54	2

EF1% = enrichment factor; BEDROC160.9 = Boltzmann-enhanced discrimination of receiver operating characteristic; A = hydrogen bond acceptor; H = hydrophobic; R = aromatic ring.
