# Supplementary material for: Combining the Classification and Pharmacophore Approaches to Understand Homogeneous Olfactory Perceptions at Peripheral Level: Focus on Two Aroma Mixtures
Source: Molecules. 2023 May 11;28(10):4028. doi: 10.3390/molecules28104028 (PMC10221229; doi:10.3390/molecules28104028)
Supplement: Supplementary file 1 [file molecules-28-04028-s001.zip › Table S4.pdf]

Table S4. Distances between features of the hypotheses hyp-V-c, hyp-IA-c, hyp-F-c, hyp-EA-c, hyp-bD-c, hyp-bI-c, and hyp-WL-c.

Hypothesis	Feature 1	Feature 2	Distance (Å)
hyp-V-c	A1	A2	5.267
	A1	H5	1.424
	A3	A1	2.742
	A3	A2	6.452
	A3	H5	4.165
	A3	R6	2.767
	H5	A2	5.167
	R6	A1	2.796
	R6	A2	3.735
	R6	H5	3.734
hyp-IA-c	A1	H3	4.287
	A1	H4	1.828
	A2	A1	2.303
	A2	H3	5.815
	A2	H4	3.558
	H4	H3	2.468
hyp-F-c	A2	A1	8.621
	A2	R4	2.758
	R4	A1	5.967
hyp-EA-c	A2	A1	2.303
	H3	A1	1.826
	H3	A2	3.554
hyp-bD-c	A1	H2	3.353
	A1	H3	3.225
	A1	H4	3.853
	A1	H5	3.714
	H2	H3	2.584
	H2	H4	5.085
	H2	H5	2.886
	H3	H4	4.698
	H3	H5	2.501
	H4	H5	2.974
	H6	A1	3.616
	H6	H2	5.267
	H6	H3	6.397
	H6	H4	4.576
	H6	H5	5.662

hyp-bI-c	A1	H2	5.082
	A1	H3	5.464
	A1	H4	5.112
	A1	H5	5.803
	H2	H3	2.576
	H2	H4	4.980
	H2	H5	2.846
	H3	H4	4.788
	H3	H5	2.574
	H5	H4	2.964
hyp-WL-c	A1	H4	4.273
	A1	H5	1.781
	A2	A1	2.255
	A2	H4	6.392
	A2	H5	3.730
	H3	A1	3.686
	H3	A2	4.888
	H3	H4	5.864
	H3	H5	2.361
	H5	H4	3.869
