# Supplementary material for: Combining the Classification and Pharmacophore Approaches to Understand Homogeneous Olfactory Perceptions at Peripheral Level: Focus on Two Aroma Mixtures
Source: Molecules. 2023 May 11;28(10):4028. doi: 10.3390/molecules28104028 (PMC10221229; doi:10.3390/molecules28104028)
Supplement: Supplementary file 1 [file molecules-28-04028-s001.zip › Table S5.pdf]

Table S5. Details of the PHASE top ten hypotheses generated from V-s, IA-s, F-s, EA-s, bD-s, bI -s and W-s subsets.

Subset	Hypothesis	Phase Hypo Score	EF1%	BEDROC160.9	Ranked Actives
V-s	AHR_3	1.14	84.33	0.97	12
	AHR_1	1.11	75.90	0.91	12
	AHR_2	1.03	75.90	0.89	11
	AAR_2	1.03	75.90	0.89	10
	AAR_1	1.03	84.33	0.97	11
	AAR_3	1.02	75.90	0.89	10
	AAHR_1	0.91	75.90	0.89	9
	AAHR_3	0.90	75.90	0.89	10
	AAAR_2	0.90	75.90	0.89	9
	AAAR_1	0.90	75.90	0.89	9
IA-s	AAH_1	1.26	91.91	0.99	11
	AAH_3	1.25	91.91	0.98	11
	AAH_2	1.17	91.91	0.96	10
F-s	AAR_1	0.93	70.70	0.84	7
	AAR_2	0.92	70.70	0.82	7
	AHR_2	0.74	50.50	0.59	7
	AAH_1	0.72	50.50	0.61	7
	AAHR_1	0.72	50.50	0.67	5
	AHR_5	0.71	50.50	0.67	6
	AHR_3	0.71	50.50	0.69	7
	AHR_1	0.69	40.40	0.56	7
	AAHR_4	0.69	40.40	0.56	6
	AAHR_2	0.69	40.40	0.61	6
EA-s	AAH_1	1.16	78.48	0.92	8
	AAH_2	1.13	78.48	0.89	8
bD-s	HHH_1	1.10	71.93	0.76	7
	AHH_2	1.07	71.93	0.79	7
	AHH_3	1.06	71.93	0.82	7
	AHHH_1	1.06	71.93	0.77	7
	AHHH_5	1.04	86.31	0.82	7
	AHH_1	0.96	71.93	0.82	7
	AHHH_2	0.93	57.54	0.74	7
	HHH_2	0.92	71.93	0.82	7
	AHHH_3	0.92	57.54	0.73	7
	AHHH_4	0.91	57.54	0.69	7

Subset	Hypothesis	Phase Hypo Score	EF1%	BEDROC160.9	Ranked Actives
bI-s	AHH_3	1.20	89.69	0.95	9
	HHH_2	1.14	89.69	0.95	8
	HHH_1	1.14	89.69	0.94	8
	AHH_2	1.14	89.69	0.94	9
	AHH_1	1.14	89.69	0.95	8
	AHHH_9	1.13	78.48	0.89	8
	HHH_3	1.08	78.48	0.88	8
	AHH_4	1.07	67.27	0.82	9
	AHH_6	0.99	67.27	0.81	9
	AHH_5	0.98	67.27	0.81	9
WL-s	AAH_1	1.08	88.20	0.85	7
	AHH_1	1.02	63.00	0.69	8
	AHH_2	0.99	75.60	0.83	7
	AHH_3	0.94	50.40	0.70	8
	AAHH_1	0.89	63.00	0.75	6
	AAH_2	0.80	37.80	0.54	6
	AHH_4	0.78	50.40	0.64	8
	AAH_3	0.62	25.20	0.40	7
	HHH_1	0.61	25.20	0.32	5
	AHH_5	0.52	25.20	0.32	8

EF1% = Enrichment factor; BEDROC160.9 = Boltzmann-enhanced discrimination of receiver operating characteristic; A = hydrogen bond acceptor; H = hydrophobic; R = Aromatic ring.
