# Supplementary material for: Combining the Classification and Pharmacophore Approaches to Understand Homogeneous Olfactory Perceptions at Peripheral Level: Focus on Two Aroma Mixtures
Source: Molecules. 2023 May 11;28(10):4028. doi: 10.3390/molecules28104028 (PMC10221229; doi:10.3390/molecules28104028)
Supplement: Supplementary file 1 [file molecules-28-04028-s001.zip › Table S6.pdf]

Table S6. Distances between features of the hypotheses generated from V-s, IA-s, F-s, EA-s, bD-s, bI-s, and WL-s.

Subset	Feature 1	Feature 2	Distance (Å)
Hyp-V-s	A3	R6	2.770
	H5	A3	4.146
	R6	H5	3.579
hyp-IA-s	A1	H3	2.406
	A2	A1	2.274
	H3	A2	3.707
hyp-F-s	A1	R4	3.594
	A2	A1	2.274
	R4	A2	5.367
hyp-EA-s	A1	H3	2.791
	A2	A1	2.274
	H3	A2	3.900
hyp-bD-s	H4	H6	2.835
	H6	H7	5.102
	H7	H4	4.368
hyp-bI-s	A1	H5	6.169
	H4	A1	4.125
	H4	H5	2.926
hyp-WL-s	A1	A2	2.309
	A2	H5	4.557
	H5	A1	2.767
