# Supplementary figures and images for: Combining the Classification and Pharmacophore Approaches to Understand Homogeneous Olfactory Perceptions at Peripheral Level: Focus on Two Aroma Mixtures
Source: Molecules. 2023 May 11;28(10):4028. doi: 10.3390/molecules28104028 (PMC10221229; doi:10.3390/molecules28104028)

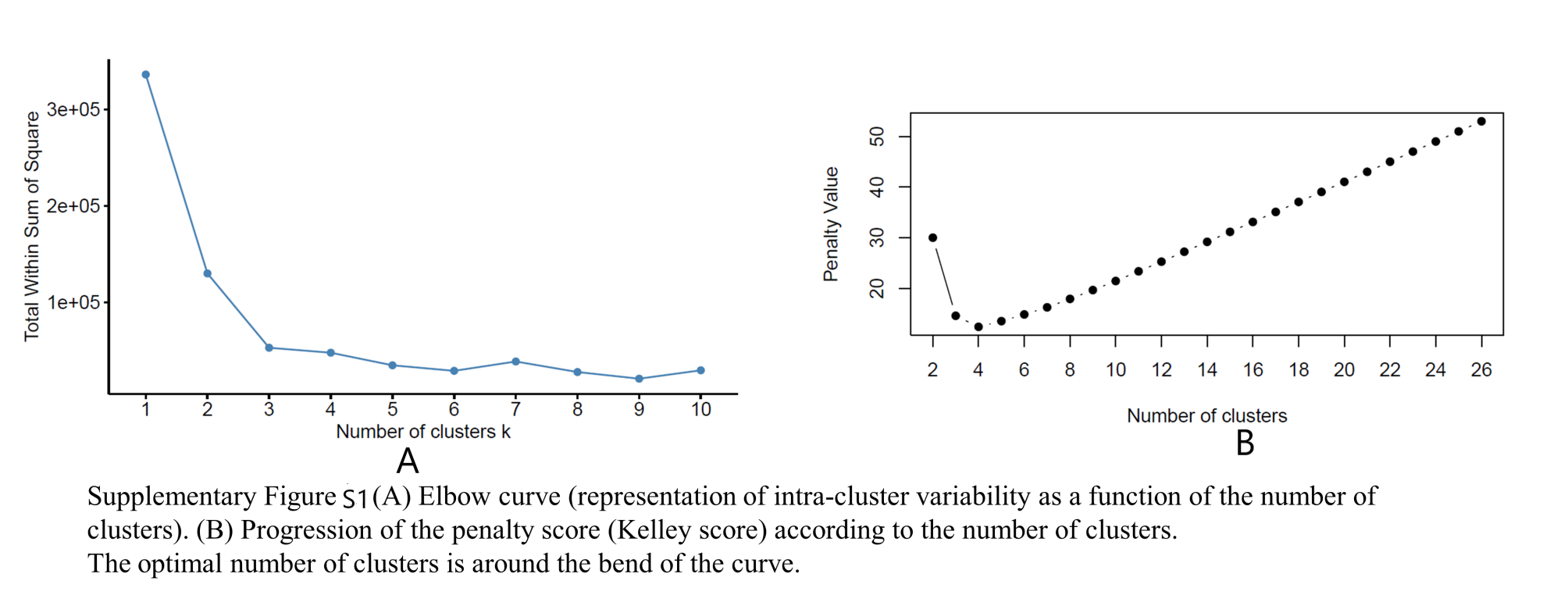

Supplement: Supplementary file 1 [file molecules-28-04028-s001.zip › Figure S1.tif]
